# A Secure Communication Suite for Underwater Acoustic Sensor Networks

**DOI:** 10.3390/s121115133

**Published:** 2012-11-07

**Authors:** Gianluca Dini, Angelica Lo Duca

**Affiliations:** 1 Department of Ingegneria dell'Informazione, University of Pisa, Largo Lazzarino 1, 56100 Pisa, Italy; 2 Institute of Informatics and Telematics, National Research Council, Via Giuseppe Moruzzi 1, 56124 Pisa, Italy; E-Mail: angelica.loduca@gmail.com

**Keywords:** underwater acoustic sensor network, security, confidentiality, integrity, secure routing

## Abstract

In this paper we describe a security suite for Underwater Acoustic Sensor Networks comprising both fixed and mobile nodes. The security suite is composed of a secure routing protocol and a set of cryptographic primitives aimed at protecting the confidentiality and the integrity of underwater communication while taking into account the unique characteristics and constraints of the acoustic channel. By means of experiments and simulations based on real data, we show that the suite is suitable for an underwater networking environment as it introduces limited, and sometimes negligible, communication and power consumption overhead.

## 1. Introduction

Underwater Acoustic Sensor Networks (UASNs) are constituted by underwater sensor nodes and autonomous underwater vehicles (AUVs) interconnected by a wireless acoustic underwater communication network. UASNs can be used for many purposes including ocean sampling, environment monitoring, undersea explorations, distributed tactical surveillance and disaster prevention [1].

Co-ordination and sharing of information among sensor nodes and AUVs require secure communication [2]. Since the acoustic channel is an open medium, an attacker conveniently equipped by an acoustic modem can easily eavesdrop on the messages traversing the network. This could be very dangerous, for example, in distributed tactical surveillance applications where messages must be secret. Furthermore, the attacker can also modify or inject fake messages so compromising the integrity of the system at both the application and the network layer. The level of damage that may ensue depends on the specific case. However, it may include an alteration of the positives/negatives rate, forms of denial of service deriving from the violation of the integrity of the routing service [3], and even the loss or the damage of AUVs and sensor nodes. All these considerations show the urgency of establishing secure channels among underwater nodes.

The unique characteristics of the underwater acoustic channel, and the differences between UASNs and their ground counterpart, namely Wireless Sensor Networks (WSNs) [4], require the development of efficient security mechanisms. Radio waves do not propagate well underwater due to the high energy absorption of water. Therefore, underwater communication is based on acoustic waves that are characterised by large propagation delays. The propagation speed of sound in water is typically 1,500 m/s, five orders of magnitude lower than light speed. In addition, acoustic links have low bandwidth and low quality due to the chemical-physical properties of the water medium such as temperature, salinity, density and spatio-temporal variations. Furthermore, underwater hardware is more expensive than WSNs nodes, and thus underwater nodes are sparsely deployed. It follows that underwater communication have more stringent power requirements than terrestrial systems because acoustic communication are more energy expensive, distances between underwater nodes are greater and thus higher power is required to ensure coverage [1].

In this paper we present the secure communication suite for UASNs we have designed and experimented within the European project called “Underwater Acoustic Network” (UAN) [5]. We have considered a UASN composed of both AUVs and fixed underwater sensor nodes that cooperate towards the same application. Nodes may move as well as dynamically join and leave the system. A node joins the system when its mission starts and leaves when its missions finishes. A node may also be forced to leave when suspected of being compromised. The reference application scenario was a distributed surveillance system. The secure communication suite is composed of two integrated components—a *secure routing protocol* and a set of *cryptographic primitives* (cipher, digest, and re-keying)—and aims at protecting confidentiality and integrity of the application and network control messages while taking into account the peculiarities of the underwater acoustic medium.

The paper provides the following novel contributions. First of all, the paper presents a practical and efficient solution to secure communication in UASNs. The proposed suite (i) protects end-to-end confidentiality and integrity; (ii) supports both the one-to-one and one-to-many communication paradigm; (iii) allows secure reconfiguration due to nodes mobility, joining, and leaving. The resulting suite introduces limited communication overhead and negligible energy consumption. We show that these claims are well founded by means of sea trials and simulations based on real data. To the best of our knowledge, this is the first secure communication suite that has been implemented and tested in the field.

Second, by means of experiments and real data, the paper proves that the resulting security suite is indeed suitable for an underwater acoustic networking environment. We believe that reporting design choices and field performance is valuable in itself because it may allow the research community to orient towards either refinements of the proposed approach or different approaches altogether, however with the long-term objective of achieving operational implementations of secure underwater acoustic networks.

The paper is organized as follows. Section 2 discusses related work. Section 3 and Section 4 describe the system and threat model, respectively. Section 5 describes the cryptographic suite whereas Section 6 describes the secure routing. Section 7 presents performance evaluation carried out by means of experiments in the field and simulations with real data. Finally, Section 8 reports our final considerations.

## 2. Related Work

The problem of secure underwater acoustic communication is quite a new research field [1,2]. Domingo presents a survey of security issues, and possible countermeasures. Similarly, [6,7] analyze the security and threats of UASN. Dong *et al.* make a taxonomy of the attacks against a UASN [8]. They classify the attacks into three categories: (a) physical attacks against nodes; (b) attacks against the network; and finally, (c) attacks against the protocols. Although they give guidelines to contrast such attacks, they do not propose any concrete solution.

Some solutions to specific security problems have been presented. Dong *et al.* have proposed mechanisms for network reconfiguration when one or more nodes get destroyed [9,10]. Solutions against wormholes have been presented in [11,12]. Zuba *et al.* study the effects of denial-of-service jamming attacks on UANs using real-world field tests by building their own hardware jammer [13]. They show that underwater acoustic networks can be easily jammed by carefully using timed attacks, which are energy efficient. Finally, Liu and Zhang have proposed a key establishment scheme that is based on the channel characteristics [14] whereas Liu *et al.* have proposed a key distribution scheme based on the mobility model of underwater mobile sensors [15].

In this paper, we face with an orthogonal problem, namely secure end-to-end communication. We present a secure communication suite that: (i) protects end-to-end communication; (ii) supports both the one-to-one and one-to-many communication model; (iii) allows secure network reconfiguration due to nodes mobility, joining and leaving; and finally, (iv) has been evaluated in the real-world field. To the best of our knowledge, no such a contribution has been given so far. Cong *et al.* have proposed a cross-layer scheme for secure communication but they do not go further than illustrating the basic ideas underlying their proposal [16]. In particular, they do not provide an implementation and a performance evaluation.

The communication suite is composed of two components, a set of cryptographic primitives and a secure routing protocol. As to the cryptographic primitives, they have been initially proposed in [17]. Then, in [18] we have employed them to protect application-level communications. Here, we present the integration of the primitives with the routing protocol and show that they are adequate at the network-layer too. As to the secure routing protocol, the basic ideas behind it have been sketched in [19]. Here, we provide a complete and detailed description of the protocol as well as an evaluation of its performance.

## 3. System Model

We consider an underwater surveillance system aimed at protecting a critical asset by means of an underwater acoustic network. The system is composed of a set of networked underwater nodes, each equipped with sensing, computing and acoustic communication facilities. Every underwater node has computational resources and power saving capabilities comparable to those of a low-budget personal computer (e.g., a PC/104 embedded computer) and it is able to run a commodity operating system such as Windows XP or Linux. Nodes are battery operated and thus have limited energy resources.

Every node is equipped with a number of sensors that allow it to both sense the state of surrounding waters and detect the presence of targets in the neighbourhood. Of course, a node may only achieve a local partial view of the state of the system. For this reason, several nodes are deployed around the critical asset and each of them reports the sensed data to a Command and Control Center (C3) station. Such a station collects sensed data from underwater nodes, so achieving a global view of the system (e.g., it performs the intrusion detection algorithm).

Underwater nodes may be both fixed and mobile. Mobile nodes are *autonomous underwater vehicles* (AUVs). AUVs are crucial components in the surveillance system. Intuitively, the surveillance system strives to cover the largest connected detection area. Due to the changing sea conditions, fixed nodes alone would be hardly able to guarantee this requirement all the time. For this reason, the system employs AUVs that dynamically change their position in order to fulfill the requirement. AUVs move to target locations in response to specific commands from the C3 station. Upon reaching their respective target locations, AUVs dynamically and cooperatively adjust their positions according to the changed sea conditions in order to maintain communication and detection connectivity. We do not provide any greater details about the application and the co-operation algorithm because here we are interested in the communication model the application subsumes. The communication model will be the starting point for specifying the requirements of secure communication. More details about the application and the cooperation algorithms can be found in [20].

**Figure 1.**
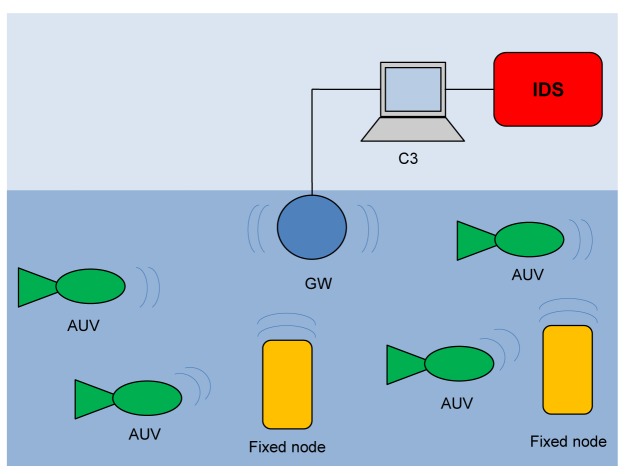
The surveillance network.

The above system model indicates two different communication paradigms that we need to support: (a) *one-to-one* communication between C3 and a single underwater node; and (b) *one-to-many* communication among nodes. Given the peculiarities of an underwater communication [1], we support these paradigms by means of a network organized as shown in Figure 1.

**Figure 2.**
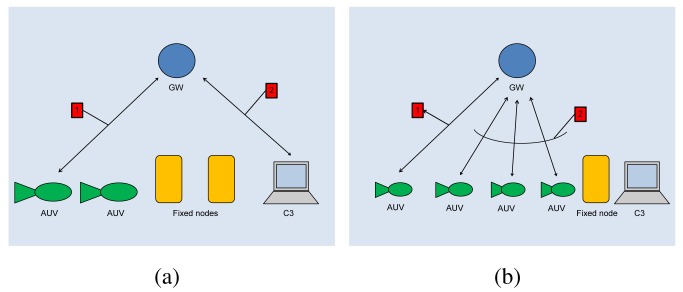
Implementation of the (**a**) one-to-one and (**b**) one-to-many communication paradigms.

The *gateway* (GW) is a node located close to the asset and capable of both radio and acoustic communication. The gateway is not energy constrained as it receives power from the asset through a power cable. In the one-to-one communication paradigm, the gateway GW relays messages between underwater nodes and the C3 station (see Figure 2(a)).

As to the one-to-many communication, in principle a node could directly broadcast a message to the destinations. However, as the power necessary to send a message decays with a power of distance greater than two [1,21], then the power necessary to reach all destinations would be too large, and nodes would drain their battery very quickly. For this reason, with reference to Figure 2(b), when a node wishes to broadcast a message, it actually sends the message to the GW (message 1) which actually broadcasts it (message 2). The gateway is not power constrained and thus it can use all the necessary power.

## 4. Threat Model

An underwater acoustic network is subjected to several threats and possible attacks [16]. An adversary equipped with an acoustic modem can eavesdrop on messages as well as modify and inject fake messages so endangering the confidentiality and integrity of application messages.

In a surveillance system, integrity is as important as confidentiality. Actually, an adversary that is able to spoof a node and thus impersonate it can then inject fake messages. Fake messages may carry wrong measurements as well as wrong commands for mobile nodes. Wrong measurements may lead the surveillance system to return wrong results in terms of false positives/negatives. Wrong commands may cause the network to disrupt or even translate into a safety issue with possible damages to devices themselves.

In order to protect integrity and confidentiality, we assume that underwater communications are enciphered. At the application level, every node *u* shares a secret symmetric *end-to-end* key, *e_u_* with the gateway GW for one-to-one communication. Node *u* uses the key *e_u_* to encrypt unicast application message addressed to GW, and to decrypt unicast messages received from GW.

In addition, in order to support one-to-many communication, the gateway GW and the mobile nodes are organized as a *group* and, as such, they share a secret symmetric *group key g*. Every group member uses *g* to encrypt/decrypt application messages before broadcasting/receiving them into/from the group.

An adversary may also attack availability. Denial of Service (DoS) is a severe threat in an underwater acoustic network, even more severe than in traditional networks, due to the intrinsic limitations of the acoustic channel [21]. In the UAN project, we have contrasted DoS at multiple different layers. In this paper we mainly focus on DoS that may derive from attacks to network integrity [3]. We come back to this point later in Section 6 for more details. However, here, we would like to spend just a few intuitions about the countermeasures against DoS we have adopted at the application level. These countermeasures are complementary to those applied at the network layer. The cooperative algorithm that allows UAVs to maintain the largest sensing area is intrinsically reactive against DoS due to the *emergent behaviour* approach on which it is based [22,23]. In brief, whenever an adversary succeeds in disrupting communication among vehicles, the simple rules that drive vehicles motion make them move closer to the asset in order to ensure protection regardless what the other agents are doing. As a result, a DoS attack effectively degrades the performance of the cooperation, but it cannot prevent continuing the surveillance mission with a limited number of cooperating nodes, or even with all the nodes acting individually. A detailed description of the algorithm can be found in [20]. Furthermore, from a practical standpoint, if the DoS attack persists, an underwater node may take an extreme measure, namely surface and communicate with a land station using standard radio communication technologies. We have used this approach in a surveillance system but it can be generalized to other underwater application scenarios.

## 5. The Cryptographic Suite

Supporting secure communication in an underwater acoustic network is challenging due to the severe limitations of the networking environment in terms of very high message propagation delay, very low bandwidth, and high energy consumption for communication [21].

Cryptography is the primary means to support confidentiality and integrity. Cryptographic primitives tend to cause the *ciphertext expansion* phenomenon. Actually, the message padding as well as modification identification codes (MICs) and message authentication codes (MACs) make the message length to increase after applying cryptography [24]. It follows that message expansion becomes responsible of a longer transmission delay and an increased energy consumption. Depending on the cryptographic primitive, such an additional communication overhead due to security may be comparable to, if not even greater than, the overhead related to transmitting the payload itself. It follows that a properly designed cryptographic suite for an underwater acoustic network has to avoid, or at least mitigate, the message expansion phenomenon.

Furthermore, underwater nodes are generally deployed in unattended, possible hostile, environments [25]. Generally, cost reasons prevent us to deploy tamper-resistant devices. For this reason, we cannot exclude that an adversary may be able to compromise an underwater node and alter its behaviour. Detecting compromised nodes much depends on detecting anomalies in expected communication and movement patterns [26], which are highly application-dependent. However, reactive measures to remove the compromised node from the system once detected should be considered by design. Concerning this, the cryptographic suite provides rekeying as a reactive measure to remove, at least logically a compromised device from the network. Actually, by revoking the keys held by a compromised device we revoke its ability to send and receive messages. However, in some situations revoking keys can be insufficient. Indeed the intruder could still be able to flood or jam the network. In these cases, physical removal becomes essential. However, even in situations where physical revocation of a compromised node is necessary, the ability of rekeying the system is indispensable. Actually, although the misbehaving device will be physically removed, it has been under the adversary control for a while and thus any secret therein contained (e.g., passwords and cryptographic keys) cannot be trusted anymore and have to be considered compromised.

In Sections 5.1 and 5.2 we describe our solutions aimed at avoiding message expansion in secure message communication. In Section 5.3 we describe an efficient and scalable rekeying service.

### 5.1. Confidentiality

Confidentiality of messages is achieved by encrypting them with a block cipher. Encryption is achieved by splitting cleartext in blocks of fixed, predefined bit-length and then encrypting every block. In the most general case, cleartext length is not multiple of the block length. Thus padding is necessary. However, padding has the negative effect that the ciphertext may result up to one block longer than the corresponding cleartext. This effect is an instance of the ciphertext expansion phenomenon.

While ciphertext expansion is negligible in a traditional network, it becomes relevant in wireless sensor networks and, in particular, underwater acoustic networks. In these networks, communication and energy limitations require to keep a message size small and ciphertext expansion may introduce an overhead that is not negligible anymore. For instance, in the AES encryption algorithm the block size is 128 bits. As the message size in the UAN project is, on average, around 720 bits, then the message expansion due to padding would be around 18%.

In order to completely avoid the ciphertext expansion problem, we use the CipherText Stealing (CTS) technique that alters the processing of the last two blocks of plaintext, resulting in a reordered transmission of the last two blocks of ciphertext and no ciphertext expansion [27].

### 5.2. Integrity

Encryption without authentication is insecure [24]. For example, an adversary may flip bits in unauthenticated ciphertext and cause predictable changes in the plaintext that receivers are not able to detect. To address this vulnerability, the system always authenticates messages. Security of hash functions is directly related to the length of the digest. However, as a digest is appended to the message, it becomes another source of message expansion and consequent communication overhead. For instance, the size of a digest produced by SHA-256 is 256 bits, which causes an additional overhead that amounts to around 35% of an average UAN message.

UAN features a trade-off between security and performance by using 4 bytes digests resulting from truncating the real hash function value. Using, such a short hash size, the overhead becomes around 4.4% of the UAN average message, which is almost negligible. Furthermore, using such a short hash function value is not detrimental to security [28]. An adversary has 1 in 2^32^ chances to blindly forge a digest. If an adversary repeatedly tries to forge it, he/she needs at maximum 2^31^ trials. However, the adversary cannot perform trials off-line. This means that the adversary has to validate a given forgery only by sending it to an authorized receiver. This implies that the adversary has to send 2^31^ messages in order to successfully forge a single malicious message. In a conventional network, this number of trials is not large enough. However, in an underwater acoustic network this may provide an adequate level of security. An adversary can try to flood the network with forgeries, but on a 500 bps channel with 184-bit messages, he/she can only send about 2.71 attempts for second. Thus, sending 2^31^ messages requires around 306 months, *i.e.*, about 25 years. Battery-operated vehicles have not enough energy to receive that many messages. Furthermore, the integrity attack would translate into a denial of service attack since the adversary needs to occupy the acoustic channel for a long time. Fortunately, it is feasible to detect when such an attack is underway. UAN uses a simple heuristic: vehicles could signal the base station when the rate of digest/MAC failures exceeds some predetermined threshold.

### 5.3. Group Key Management

Each time a node leaves the system, the GW generates and distributes a new group key *g*. This is done to avoid that an old node is able to read new messages. As to rekeying protocol, we chose S2RP, a secure and scalable rekeying protocol for resource-constrained devices [29,30]. S2RP is particularly suitable for UAN for two reasons. First of all, S2RP provides a very efficient proof of key authenticity. Actually, S2RP verifies the authenticity of a key by computing a digest of it. So, verification is very computing efficient and does not require any additional information, e.g., MACs or digital signatures, which would cause a great message expansion. Secondly, S2RP requires a number of rekeying messages that is logarithmic in the number of nodes so making the key distribution phase highly scalable.

**Figure 3.**
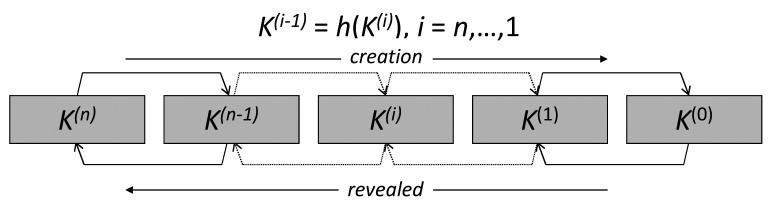
Key chain.

In short, the key authentication mechanism levers on *key-chains*, a technique based on Lamport's one-time passwords. A key-chain is a set of symmetric keys so that each key is the hash pre-image of the previous one (see Figure 3). Hence, given a key *k*^(^*^i^*^)^ in the key-chain, anybody can compute all the previous keys *k*^(^*^j^*^)^, *j* ≤ *i*, however nobody, but the key-chain creator, can compute any of the next keys *k*^(^*^j^*^)^, *j* > *i*. Keys are revealed in the reversed order with respect to creation order. Therefore, given an authenticated key in the key-chain, anybody can authenticate the next revealed keys by simply applying a hash function. For example, if *k*^(^*^i^*^)^ is an authenticated key, than anyone can verify the authenticity of *k*^(^*^i^*^+1)^ by verifying that *k*^(^*^i^*^)^ = *h*(*k*^(^*^i^*^+1)^). We call *current key* of a key-chain the last revealed key of the key-chain and *next key* the hash pre-image of that key. We denote them by *k* and *k*^+^, respectively. Notice that *k* = *h*(*k*^+^).

**Figure 4.**
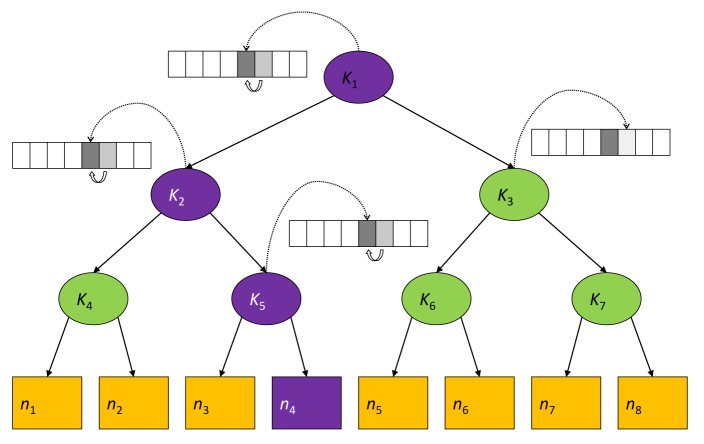
Key tree.

To reduce the communication overhead, the gateway GW maintains a logical key tree (see Figure 4) where each internal node is associated to a key-chain and each leaf is associated with a node's node-key (*i.e.*, the secret key that the node shares with GW). We denote by *k_i_* the current key of the hash-chain associated with the internal tree node *i*. Keys in the hash chain are only used in the rekeying process and thus are called *key encryption keys* (KEKs).

Each node maintains a *key-ring* that contains every KEK *k_i_* such that the sub-tree rooted at node *i* contains the leaf associated with the node-key. Hence, with reference to Figure 4, the *key-ring* of node *υ*_4_ is {*k*_1_, *k*_2_, *k*_5_}. As it turns out, key *k*_1_, associated to the key tree root, is shared by all nodes and thus can act as the group-key, *i.e.*, *g* ≡ *k*_1_.

In order to have an idea of how the protocol works, let us consider the logical key tree in Figure 4. Let us now assume that node *υ*_4_ leaves the group. All keys in its key ring are considered compromised and GW has to broadcast the respective next keys $k_{1}^{+}$, $k_{2}^{+}$, $k_{5}^{+}$ by means of the following rekeying messages:

1. GW → *υ*_3_: $E_{e_{\upsilon_{3}}}(k_{5}^{+})$
2. GW → *υ*_3_: $E_{k_{5}^{+}}(k_{2}^{+})$
3. GW → {*υ*_1_, *υ*_2_}: $E_{k_{4}}(k_{2}^{+})$
4. GW → {*υ*_1_, *υ*_2_, *υ*_3_}: $E_{k_{2+}}(k_{1}^{+})$
5. GW → {*υ*_5_, *υ*_6_, *υ*_7_, *υ*_8_}: $E_{k_{3}}(k_{1}^{+})$

Upon receiving a rekeying message, after it has been properly decrypted, the authenticity of the next key therein contained is verified by computing its hash and comparing the result to the corresponding current key. For instance, upon receiving rekeying message 5, *n*_6_ decrypts the message by means of *k*_3_ and verifies the authenticity of $k_{1}^{+}$ by ascertaining that $k_{1}=h(k_{1}^{+})$. As it turns out the rekeying protocol requires *O*(log *n*) rekeying messages, where *n* is the number of nodes. Furthermore, given the key-chain authentication mechanism, every rekeying message needs to carry only the next key (in its encrypted format). No additional information proving key authenticity is thus required. Notice that this is a great advantage in terms of communication overhead with respect to using digital signatures, for example. To fix ideas, let us suppose that group keys are 128-bit long and we use ECC-180 digital signature to authenticate them. ECC-180 is nowadays considered as secure as RSA-1024. In ECC-180 a digital signature is 360 bits and thus a rekeying message would be 488 bits, *i.e.*, 3.8125 times longer than in the approach proposed here.

## 6. On Control Messages Protection

So far, our focus has been on protecting the integrity and confidentiality of application messages, namely, messages whose payload contains data sent by the application layer of the network, such as sensor readings or commands to AUVs. However, unless the surveillance communication sub-system fits within a single acoustic broadcast domain, a multi-hop, ad-hoc acoustic network is necessary. In such a case, we have to consider integrity and confidentiality of *control messages* too, namely messages whose payload contains data used to maintain the network services. These include, for instance, routing discovery messages and routing maintenance messages. All of these control messages are of fundamental importance for availability. However, contrary to confidentiality and integrity, we can not assume end-to-end security for control messages because the network is by definition a distributed protocol.

Disrupting the functionality of the routing protocols compromises the availability of the network and services running on the network. Attacks against routing protocols include attempts to create routing loops or black holes (when attacker claims to be a short distance to all destinations and then selectively forwards payload traffic). Therefore, the same principles of service integrity and service confidentiality apply to control messages. To provide a secure communication infrastructure, control messages need to have authentication and replay protection. Therefore, these messages need to use message integrity codes. Also, to prevent an outsider from identifying the type of control messages being transmitted, we should have these messages encrypted.

In the UAN project we have used FLOOD, a routing protocol that has been designed for, and tested in, an underwater acoustic networks [31]. Unfortunately, FLOOD does not provide any mechanism to protect the confidentiality and integrity of control messages and this makes FLOOD prone to attacks. For this reason, we have designed and implemented *secure FLOOD* (SeFLOOD), a secure version of FLOOD where every control message is protected by means of the cryptographic suite described in Section 5. In order to protect routing control messages we need link layer keys shared by pairs of neighbouring nodes. As these keys are meant to protect routing control messages, they have to be established before the routing protocol starts.

In Section 6.1 we provide a brief description of FLOOD. By way of example, in Section 6.2 we highlight FLOOD vulnerabilities and describe a spoofing attack against control message integrity that translates into Denial of Service (DoS). Finally, in Section 6.3 we describe SeFLOOD which incorporates the establishment of link-layer shared keys.

### 6.1. FLOOD: The Routing Protocol for UAN

FLOOD is a routing protocol for UAN. It is composed of three phases: the *network discovery* phase, the *data routing* phase and the *network reconfiguration* phase. In the *network discovery* phase, nodes discover one another in order to build the routing tables. In practice, each node reports to the GW all the neighbors it has discovered and their relative information (e.g., link quality, water conditions). Once received all the information, the GW builds the routing tables, one for each node, by applying the Dijkstra algorithm [32] and distributes them to the corresponding nodes. At this point the network discovery phase ends and the *data routing* phase begins. In this phase, each node routes messages according to the routing tables received from the GW. Upon a node's leaving or joining the network, a *network reconfiguration* phase is performed.

As to the security of the control messages and thus the integrity of the network infrastructure, the network discovery phase and the network reconfiguration phase are particularly relevant. For this reason, we briefly review them in Sections 6.1.1 and 6.1.2 as far as their salient features related to security are concerned. For a detailed description of FLOOD, readers may refer to [31].

#### 6.1.1. The Network Discovery Phase

**Figure 5.**
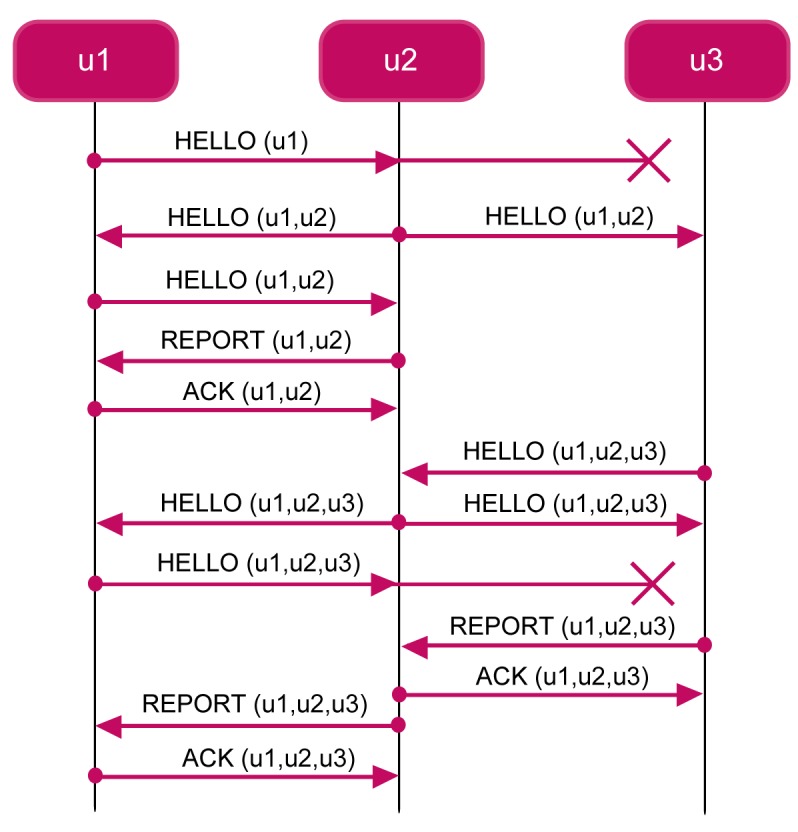
An execution instance of FLOOD.

The FLOOD Network Discovery Phase is based on the *flooding* principle. The protocol is based on the *flooding mechanism*. Upon receiving a control discovery message, a node re-broadcasts it unless the node has not already done it. To fix ideas, consider Figure 5 which shows an execution instance of the FLOOD protocol. In the figure, we show that the network is composed of three nodes. Furthermore, assume that the transmission range of node *u*_1_ (the gateway) only covers node *u*_2_, whereas node *u*_2_ is able to reach both *u*_1_ and *u*_3_, and, finally, node *u*_3_ is able to reach only *u*_2_.

The protocol is initiated by the GW which broadcasts a 
HELLO(*u*_1_), including its identifier and the time *t_i_* at which the message is sent. Upon receiving the hello message, the node *u*_2_ adds to the message its identifier and other information, including the quality of the link (e.g., signal attenuation). Then *u*_2_ relays the message 
HELLO(*u*_1_,*u*_2_) by broadcasting it after a randomized delay. When *u*_1_ receives the message, it re-broadcasts it. When *u*_2_ receives the new message, it sends to *u*_1_ a 
REPORT(*u*_1_,*u*_2_) unicast message. The GW acknowledges *u*_2_ by sending it a 
ACK(*u*_1_,*u*_2_) unicast message. With the ack message, the protocol between *u*_1_ and *u*_2_ is completed. However, also the node *u*_3_ receives the 
HELLO(*u*_1_,*u*_2_) message. Suppose that *u*_3_ receives it after that *u*_2_ receives 
ACK(*u*_1_,*u*_2_). The node *u*_3_ adds to the message its identifier and information about link quality. Then it broadcasts HELLO(*u*_1_,*u*_2_,*u*_3_). Upon receiving this message, the node *u*_2_ re-broadcasts it. The message is received both by the GW *u*_1_ and the node *u*_3_. Upon receiving the message, the node *u*_3_ builds a report message 
REPORT(*u*_1_,*u*_2_,*u*_3_). When *u*_2_ receives the report message from *u*_3_, it sends it the 
ACK(*u*_1_,*u*_2_,*u*_3_) unicast message so that the protocol between *u*_2_ and *u*_3_ is complete. When the GW receives the message 
HELLO(*u*_1_,*u*_2_,*u*_3_) it re-broadcasts it. Upon receiving the message the node *u*_2_ builds a report message REPORT(*u*_1_,*u*_2_,*u*_3_). When *u*_1_ receives the report message from *u*_2_, it sends it the 
ACK(*u*_1_,*u*_2_,*u*_3_) unicast message so that the protocol between *u*_1_ and *u*_2_ is complete.

#### 6.1.2. The Network Reconfiguration Phase

FLOOD allows the network to reconfigure, *i.e.*, it allows an AUV to move from one part of the network to another. From a network management point of view, this operation requires to: (i) reconstruct the neighbourhood of both the detaching and re-attaching points; (ii) update the routing tables. FLOOD supports this reconfiguration phase under the following assumptions: (a) each mobile node knows when it is going to move and when it has reached its new location; (b) each mobile node does not send/receive messages while moving.

Before starting moving, a mobile node *u_m_* sends GW a 
DEL(*u_m_*). Upon receiving the message, GW updates the routing tables accordingly. If some nodes become unreachable through the new routing table, GW triggers a new instance of the Network Discovery Phase. Otherwise, it can continue the Network Reconfiguration Phase. In the latter case, GW sends a BEST message to every node for which the routing table has been updated. The message includes also information about the new next hop to reach the GW.

Upon reaching its new location, the mobile node broadcasts an 
ADD(*u_m_*) message. Upon receiving the 
ADD(*u_m_*) message, each node sends back an 
ADD-ACK message containing information about link quality. The mobile node acknowledges all the received 
ADD-ACK messages by sending back unicast 
ADD-ACK messages, containing information about its link quality. Finally, the mobile node sends an 
ADD-REPORT message to the GW so that the GW can accordingly update the routing tables and re-distribute them to all the interested nodes by means of 
ADD-BEST messages.

### 6.2. A Spoofing-Based DoS Attack

**Figure 6.**
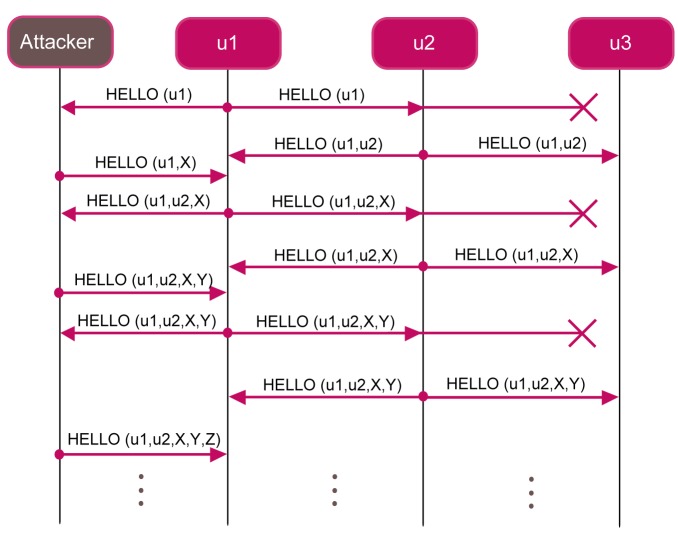
A DoS attack.

In this section, we show that FLOOD protocol is subjected to the spoofing-based DoS attack. Figure 6 shows an example of how an attacker can perform a spoofing-based DoS attack in a network composed of three nodes. Whenever the attacker receives a 
HELLO message, it can add a *new fake node* and broadcast the message to the other vehicles.

In this way, the protocol never completes. In order to avoid this kind of attack, each node could be deployed with the list of all the nodes identifiers belonging to the system. Thus, if the attacker tries to add a new fake node to the network, each node rejects it, because the fake node is not contained in that list. However, due to the underwater conditions and to the high distances among nodes, it may happen that a node *u*_1_ is not able to directly contact nodes *u*_2_ and *u*_3_ belonging to the system. The attacker could exploit this situation in order to use *u*_2_ and *u*_3_ identifier to perform a DoS attack.

### 6.3. SeFLOOD: The Secure FLOOD

In order to protect the integrity and confidentiality of the FLOOD protocol we have extended it into a new protocol called *secure FLOOD* (SeFLOOD). SeFLOOD protects control messages by establishing link-layer pair-wise keys and encrypting control messages by means of these keys. SeFLOOD uses the cryptographic suite described in Section 5.

More specifically, SeFLOOD protects confidentiality and integrity of any given control message *m* by computing *E_e_*(*m*, ⌊*h*(*m*)⌋*_t_*), where *E*(•) is a block cipher used in CBC-CTS mode (see Section 5), ⌊*h*(•)⌋*_t_* is a *t*-bits truncated hash function (see Section 5), and *e* is a symmetric key [24]. For the sake of brevity, in the following we will denote this cryptographic transformation by 〈*m*〉*_e_*. Upon receiving 〈*m*〉*_e_*, a node decrypts it by *e*, verifies the digest, and checks whether *m* contains some fresh quantity (e.g., a time stamp or a nonce) [33]. If the decryption and all the checks are successful, then we say that the node *successfully receives* the message. Otherwise, the message is discarded.

Link-layer keys are distributed as follows. We assume that each pair of nodes *u_i_* and *u_j_*, *i ≠ j,* secretly shares *a pairwise link key ℓ_ij_*, which is used to protect unicast messages between *u_i_* and *u_j_*. In order to distribute the link keys, we could use well-known key establishment schemes such as Elliptic curve Diffie-Hellman [34] or the Blundo scheme [35]. However, for both simplicity and efficiency, each node can be deployed with a *Link-Key Table* (LKT) containing all the pair-wise link keys it shares with the other nodes. This solution has an *O*(*n*) storage overhead, where *n* is the number of nodes in the system. However, it is suitable for an underwater acoustic network such as the one considered in the UAN project because the number *n* of nodes is small and each node has enough memory to store the whole Link-Key Table LKT. For example, in the case of a 128 bits link key and a network composed of 1,024 nodes, the Link-Key Table memory occupancy amounts to 16 Kbytes. As a node is typically equipped with 2 GB memory, then the storage overhead for the LKT amounts to the 0.78% of the whole memory, which is negligible.

Furthermore, we group nodes into *clusters* as follows. For each node we define a cluster that is the set of nodes belonging to the node's broadcast domain. We denote by 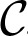
*_u_* the cluster associated with node *u.* It follows that we have as many clusters as nodes. Furthermore, a node may belong to one or more clusters. A given node *u* certainly belongs to *C_u_*. Furthermore, if *υ* is a neighbour of *u,* then *u* ∈ 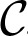
*_υ_* as well.

Each cluster is associated to a *cluster key*, which is used to secure broadcast communication among nodes belonging to the same cluster. Every node *u* generates a *cluster key c_u_* and distributes it to all the members of its cluster. Furthermore, every node maintains a *Cluster Key Table* (CKT) that has one entry for each cluster the node belongs to. Such an entry stores the cluster key of the cluster corresponding to that entry. By construction, the number of entries of the CKT is less than, or equal to, the number of entries of the LKT. This means that CKT requires a negligible amount of memory for its storage as well.

Cluster keys are established by means of the following *Cluster Key Distribution Protocol* (CKDP). Every node carries out the CKDP before initiating the FLOOD protocol. Once the link-layer cluster keys have been established, nodes use these keys to protect the integrity and confidentiality of FLOOD control messages. The Cluster Key Distribution Protocol consists of the following messages.

1. *u* → * : HELLO, *u*, *ν_u_*
2. *υ* → *u* : 〈CLUSTER_KEY, *υ*, *c_υ_*, *ν_u_*〉*ℓ_uυ_*

The protocol is composed of two rounds, the *discovery round* and the *key distribution round*. In the discovery round, a node *u* generates a fresh quantity *ν_u_* and broadcasts it together with its identifier *u* (message M6.3). Upon receiving message M6.3, a node *υ* replies *u* with its identifier *υ*, its cluster key *c_v_,* and the fresh quantity *ν_u_* (message M6.3). The message is protected by means of the link key *ℓ_uv_*. Upon successfully receiving message M6.3 from *υ* (otherwise, the message is discarded), node *u* stores the received cluster key *c_v_* into its CKT

From a security point of view, we can argue that the encipherment by *ℓ_uv_* makes node *u* to believe that *c_v_* actually comes from *υ*. Furthermore, the presence of the fresh quantity *ν_u_* guarantees that message M6.3 is not a replay of an older message. The encipherment also guarantees the secrecy of *c_v_*.

**Figure 7.**
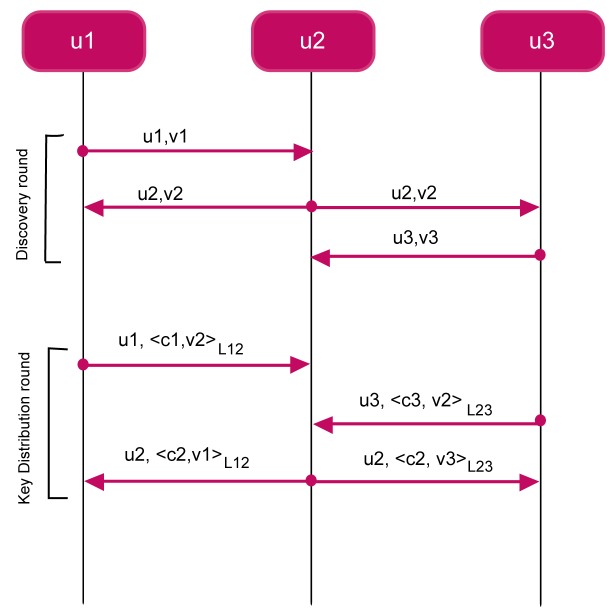
The CKDP applied to FLOOD.

Figure 7 shows an execution instance of CKDP. The network is composed of three nodes, where GW corresponds to *u*_1_. The broadcast domain of *u*_1_ covers only node *u*_2_, whereas node *u*_2_ is able to reach both *u*_1_ and *u*_3_. Node *u*_3_ is able to reach only *u*_2_. At the end of the protocol three clusters are built, 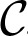
_1_= {*u*_1_, *u*_2_}, 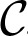
_2_ = {*u*_1_, *u*_2_, *u*_3_}, and, finally, 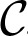
_3_ = {*u*_2_, *u*_3_} with cluster keys *c_u_*_1_, *c_u_*_2_, and *c_u_*_3_, respectively.

Notice that the attack described in Section 6.2 is not possible anymore. Actually, in order to play such an attack, the adversary would need to either know the cluster keys of neighbours or disseminate its own. However, none of these alternatives is possible because the adversary does not know the link keys of nodes.

#### 6.3.1. Secure Network Reconfiguration

SeFLOOD also supports mobile nodes, without adding overhead in terms of number of messages. In fact, it exploits FLOOD messages sent by a mobile node in order to distribute the cluster keys. We assume that nodes have loosely synchronized clocks. This assumption is reasonable because nodes periodically emerge and synchronize their clocks with GPS, for example.

Assume that *u_m_* is a mobile node wishing to leave the network. Then, it triggers the following protocol:

1. *u_m_*→ *GW*: 〈
DEL, *u_m_*, *τ_m_*〉*_e_u_m___*
2. *GW* → *u_i_*: 〈
BEST, *u_m_*, *τ_g_*, routing – updates*_i_*〉*_eui_*

Initially, *u_m_* sends the gateway a 
DEL message M6.3.1 specifying its identifier and a timestamp *τ_u_m__*. The message is protected by means of the node end-to-end key *e_u_m__*.

Upon successfully receiving message M6.3.1 (otherwise the message is discarded), the GW deletes *u_m_* from the routing tables, updates the routing tables, and sends a 
BEST message to every node whose routing table needs to be updated (message M6.3.1). The message specifies the identifier *u_m_* of the leaving node, a fresh quantity *τ_g_* and the updates to be applied to the routing table. The message is protected by means of the end-to-end key *e_i_*. Upon successfully receiving message M6.3.1 (otherwise the message is discarded), node *u_i_* updates its own routing table as specified in the message, by replacing the entries containing *u_m_* with the new ones calculated by the GW.

Assume now that the mobile node *u_m_* wants to join again the network. The node *u_m_* triggers the following protocol

1. *u_m_*→ *: 
ADD, *u_m_*, *ν_um_*
2. *u_i_*→ *u_m_*: 〈
ADD – 
ACK, *ν_um_*, *τ_i_*, *c_ui_*〉*_ℓ_im__*
3. *u_m_*→ *u_i_* : 〈
ADD – 
ACK, *τ_i_*, *c_um_*〉*_ℓ_im__*
4. *u_m_*→ GW: 〈
ADD – 
REPORT, *τ_um_*, *C_um_*〉*_e_m__*
5. GW → *u_i_*: 〈
ADD – 
BEST, *τ_i_*, routing – updates*_i_*〉*_e_i__*

Initially, node *u_m_* broadcasts an 
ADD message (M6.3.1) carrying its identifier and a nonce *ν_u_m__*. Upon receiving message M6.3.1 from *u_m_*, a node *u_i_* replies by means of an 
ADD-ACK message (M6.3.1) carrying a timestamp *τ_i_*, the node cluster key *c_ui_*, and the received nonce *ν_um_*, all encrypted with the link key *ℓ_im_*. Upon successfully receiving message M6.3.1 from *u_i_* (otherwise the message is discarded), node *u_m_* replies by sending a 
ADD-ACK message (M6.3.1) carrying the node cluster key *c_u_m__* and the timestamp *τ_i_*, all encrypted with the link key *ℓ_im_*. Then *u_m_* sends the gateway GW the members of its cluster 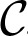
*_u_m__* (message M6.3.1). Finally the GW sends all the nodes *u_i_* (included *u_m_*) whose routing table needs to be updated an 
ADD-BEST message (message M6.3.1), carrying the updates to the respective routing table. The message contains also the timestamp *τ_i_* encrypted with *e_i_* in order to guarantee its freshness. Upon successfully receiving this message (otherwise the message is discarded), each node applies the specified updates to its own routing table.

#### 6.3.2. On Evicting a Node

In Section 5.3 we showed that a compromised node can be logically evicted from the application layer by revoking the group key and redistributing a new one to all members but the evicted one. Of course, a compromised node must be evicted at the routing layer too, as necessary. This task can be accomplished as follows. Let *u_c_* be the compromised node and *S_c_* be the set of nodes whose respective clusters contain *u_c_, i.e.*, *S_c_* = {*u|u_c_* ∈ *
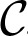
_u_*}.

In order to evict *u_c_,* the gateway GW eliminates *u_c_* from the routing tables and calculates new paths among nodes. If the new network becomes partitioned, firstly the GW informs the remaining nodes to eliminate *u_c_* from their neighbourhood, by triggering the Evicting Protocol (EP) and then it triggers the Network Discovery Phase to build new routing tables. Note that it is not necessary to trigger also the CKDP, because the neighbourhood of each node does not change. The Network Discovery Phase can be avoided when the network without *u_c_* is not partitioned.

During the EP phase, the GW sends an 
EVICT message to every node *u* in *S_c_* commanding the node to remove *u_c_* from its cluster 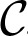
*_u_*.

$$\forall u\in S_{c},GW\to u:{\langle \text{EVICT},u_{c},\tau_{GW}\rangle }_{e_{u}}$$

where *τ_GW_* is timestamp. Upon successfully receiving the 
EVICT message (otherwise the message is discarded), node *u* removes *u_c_* from its cluster 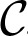
*_u_*, *i.e.*, 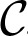
*_u_* ← 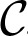
*_u_\* {*u_c_*}, generates a new cluster key $c_{u}^{\prime }$, and sends the key to every node in 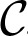
*_u_*

$$\forall \upsilon \in C_{u},u\to v:{\langle \text{CLU}-\text{KEY},u,c_{u}^{\prime },\tau_{u}\rangle }_{\ell uv}$$

## 7. Performance Evaluation

### 7.1. Performance Evaluation of the CryptoSuite

In this section we report the performance analysis of our cryptographic suite through experimental tests performed within the UAN experimental campaign conducted on May 2011 in Norway [5].

**Figure 8.**
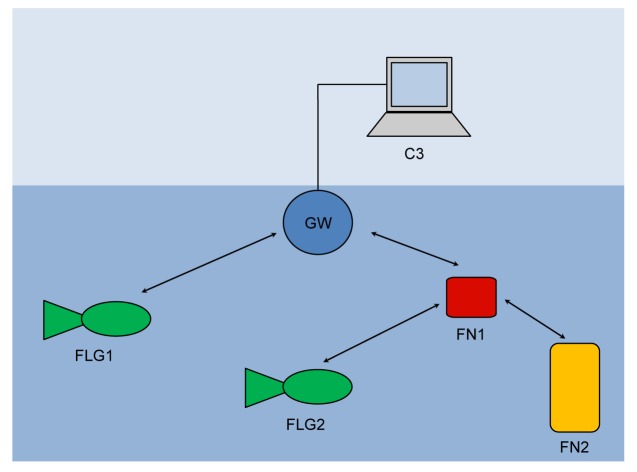
The test-bed.

**Figure 9.**
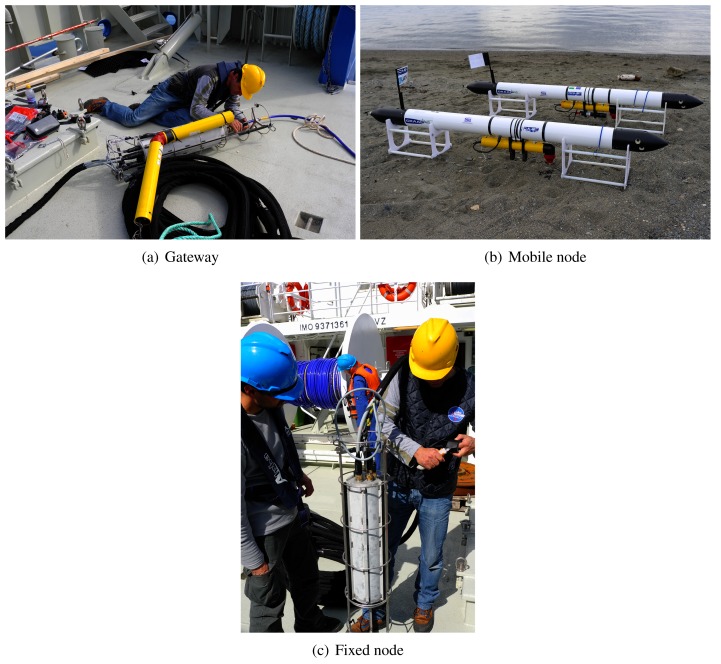
The nodes used in the experiments.

Figure 8 shows the topology of the underwater acoustic network, which constituted the testbed during the experiments. It is composed of a gateway GW, two fixed nodes (FN1 and FN2) and two UAVs (FLG1 and FLG2). Figure 9 shows the real devices we used in the experiments. Notice that the gateway (Figure 9(a)) is equal to a fixed node (Figure 9(c)) except for the optical fiber that connect the device to the asset (blue cable). UAVs are of Folaga type, manufactured by GraalTech Inc. [36] (Figure 9(b)). From a computing platform standpoint, all these computers are equipped with a 1 GHz VIA EDEN Ultra Low Voltage Processor, 1 Gbytes of RAM (DRAM DDR2 533/400 on SO-DIMM socket Chipset VIA CX700M), and run the Linux Ubuntu 10.4 Operating System. All underwater nodes are equipped with an acoustic modem developed by Kongsberg Maritime, a UAN partner, and providing a maximum transmission rate *r*_max_ 500 bits/s.

The GW is placed at a depth of 96 m. FN1 and FN2 are at a depth of 96 m and 39 m, at a distance from the GW of 163.5 m and 860 m, respectively. The nodes FLG1 and FLG2 are placed at a depth of 20 m and 15 m, with an initial distance from the GW of 677.8 m and 687.1 m, respectively. These values are summarized in Table 1.

In the experiments, we used AES in the CBC-CTS mode as cipher for confidentiality and SHA-2 as hash function for integrity. SHA-2 produces a 256-bit output that we truncate at 32 bits. It follows that guaranteeing secrecy and integrity gives rise to a message expansion of 4 bytes.

In the experiments, the payload varied in between 32 and 78 bytes. This means that the 4 bytes message size increment due to security varies in between 5.13% and 12%. This increment, of course, reflects on the increased transmission time.

Underwater acoustic communication is particularly affected by noise and subject to the highly variable sea environmental conditions. For this reason, an increased message length due to security might reflect in an increased likelihood of message loss or corruption. In order to evaluate this impact of security on performance we have considered *Average Delivery Ratio* (ADR), which is the ratio between the average number of received messages and the number of sent messages. In practice, the average delivery ratio measures the fraction of messages the network is able to deliver. Ideally, the ADR should be equal to 1.

**Figure 10.**
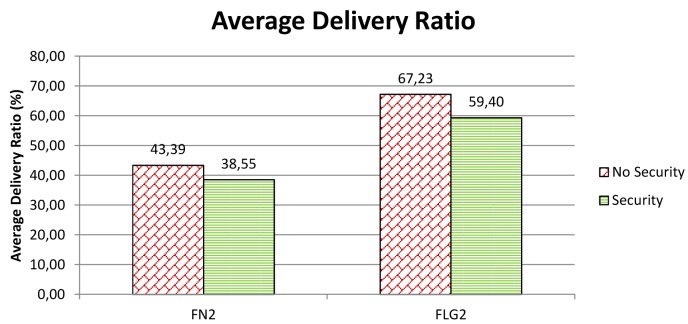
Average Delivery Ratio when security is enabled/disabled.

We have compared the ADR when security is disabled to the ADR when security is enabled. The results are reported in Figure 10. In particular, the figure shows the ADR for nodes FN2 and FLG2. We note that the ADR when security is enabled is smaller than that when security is disabled. In particular, when security is enabled, the ADR seems to suffer a decrement of 4.9% in the case of a fixed node, and 7.8% in the case of a mobile node. These variations in the ADR could be caused by an increase in the message length due to security. On the other hand, such a small percentage variations may be well depend on the change of sea conditions during the day as experiments, with security enabled and disabled respectively, were conducted at a distance of quite a few hours from each other for logistic and organizational reasons. More details about environmental conditions—e.g., water, winds, salinity, or temperatures—can be found in [5]. For these reasons, we can safely conclude that the cryptographic suite add a negligible overhead to the underwater acoustic communication.

**Figure 11.**
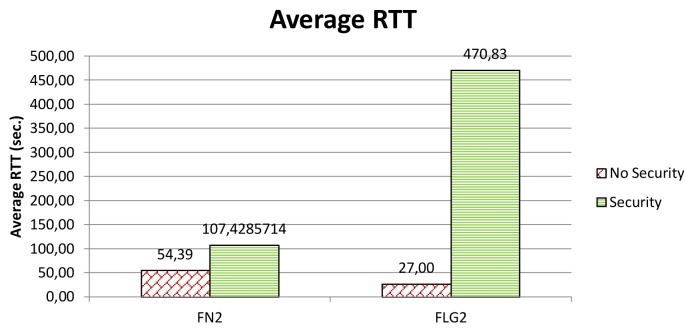
Average round-trip time when security is enabled/disabled.

In an aside, the highly variable sea conditions and their impact on the communication performance become particularly evident when you consider delay. It is well-known that underwater acoustic networks are characterized by very high delay variance which is extremely harmful for efficient protocol design, as it prevents from accurately estimating the round trip time (RTT), which is the key parameter for many common communication protocols [1]. Figure 11 shows the average round-trip time we have experienced in our experiments. Of course, the packet size increment due to integrity is not sufficient to explain the extremely large increments in the round-trip time. Actually, at a transmission rate of 500 bit/s, the additional 4 bytes would introduce just a 64 ms delay. As discussed above, the experiments with security disabled and those with security enabled were carried quite a few hours apart. In such a lapse of time, sea conditions have changed a lot so causing largely different delays.

### 7.2. Performance Evaluation of SeFLOOD

Performance of SeFLOOD has been evaluated in terms of overhead that security solutions add to FLOOD. Our evaluation encompasses both the network discovery phase and the reconfiguration phase. Such an evaluation has been carried out through a simulative approach using the SimPy simulator [37]. In the simulations we have used realistic results obtained during the field experimental campaign of the UAN project [5]. In particular, we have used an average Round Trip Time equal to about 19 s when the maximum underwater acoustic transmission rate is 500 bits/s.

**Figure 12.**
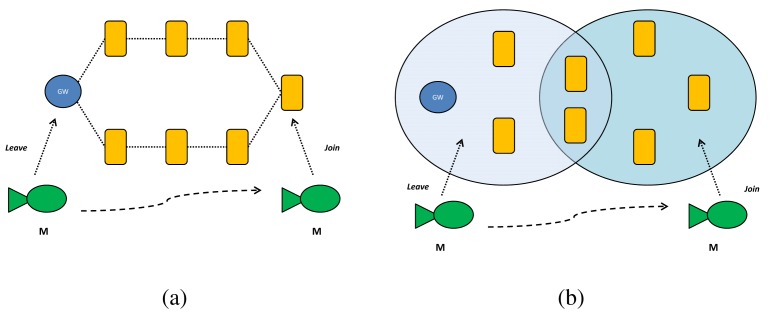
(**a**) Sparse Scenario; (**b**) Dense Scenario.

We have considered a network composed of seven fixed nodes, the gateway GW, and one mobile node. For each node we have assumed a maximum transmission power of 189 dB re *μ*Pa, a transmission range of 2 km, and a working depth of 200 m. Furthermore, we have considered two deployment scenarios aimed at evaluating performance in two different network topologies: (i) a *sparse scenario*, where each node has two neighbours; and (ii) a *dense scenario*, where each node has five neighbours (see Figure 12).

The HELLO and CLUSTER KEY messages in the Cluster Key Distribution Protocol (CKDP) have been implemented as follows. The HELLO message is composed of the following fields: (i) *msg-type* (8 bits), that specifies the message type; (ii) *node-id* (16 bits), that specifies the node identifier; (iii) *nonce* (16 bit); and, the (iv) *additional information* (24 bit), that specifies additional information (e.g., sea conditions). The CLUSTER KEY message is composed of the following fields: (i) *msg-type* (8 bits); (ii) *node-id* (16 bits); (iii) *nonce* (16 bit); the (iv) *key* (128 bit), that specifies the cluster key; and, the (v) *digest* (24 bit), that specifies the digest. It follows that the size in bits of an HELLO message is 64 (8 bytes) whereas the size of a CLUSTER KEY message is 192 (24 bytes).

We have evaluated the performance of SeFLOOD with respect to two metrics: (a) the *number of messages* (*NoM*), and (b) the *number of bits* (*NoB*). The *NoM* and *NoB* metrics measure how much communication overhead SeFLOOD adds to FLOOD in terms of total number of sent messages and total number of sent bits, respectively. Each metric *M* specifies the percentage increase of SeFLOOD with respect to FLOOD:

(1) $$M=\frac{M_{S}-M_{F}}{M_{F}}\times 100$$

where *M_S_* represents the value of the metric for the SeFLOOD protocol, while *M_F_* represents the same metric for the FLOOD protocol. Each metric has been computed as an average over fifteen runs, each lasting 5,000 s. Table 2 resumes the simulation parameters.

**Figure 13.**
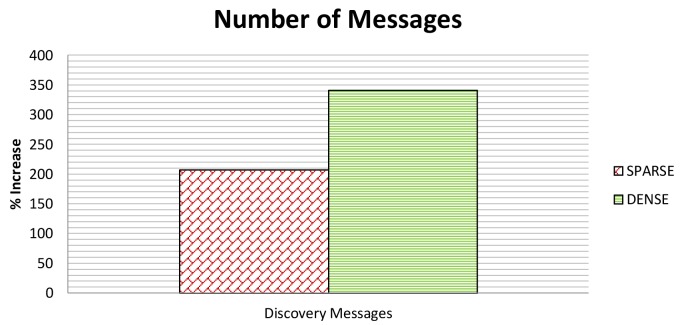
Discovery Messages in Sparse and Dense Scenarios.

**Figure 14.**
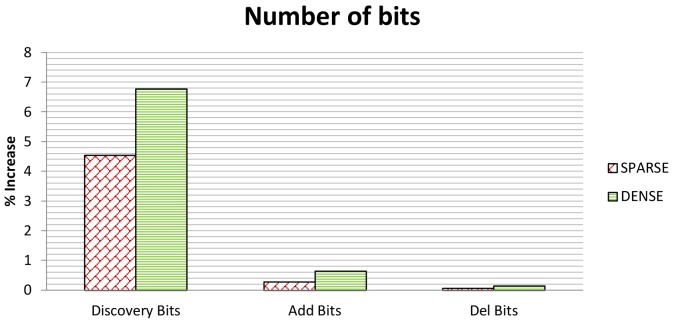
Discovery Bits, Add Bits and Del Bits in Sparse and Dense Scenarios.

Figure 13 shows the *NoM* in both the dense and sparse scenario. As to *NoM*, we only consider the discovery messages and neglect the add and delete messages because SeFLOOD does not add messages to the Reconfiguration Phase and thus SeFLOOD and FLOOD have the same number of messages in this phase. Figure 14 shows *NoB*, which, instead, considers all types of messages.

As to the Discovery Phase, the additional messages introduced by SeFLOOD derive from the Cluster Key Distribution Protocol (CKDP).

In particular, FLOOD sends 17 messages in the dense scenario and 13 messages in the sparse scenario. SeFLOOD, instead, sends 73 messages in the dense scenario and 40 messages in the sparse scenario. It follows that *NoM* is ≈ 330% in the dense scenario and ≈ 200% in the sparse scenario.

As to *NoB*, we have first to observe that the size of the CKDP messages is fixed whereas the size of FLOOD is variable. As argued above, the CKDP messages are 64 and 192 bit in size. In contrast, a FLOOD discovery message has a minimum size of 200–400 bits [31]. Furthermore, as it carries both list of the nodes through which the message has propagated and the link quality report, the message length increases with the message propagation (Section 6.1.1). By simulations, we have evaluated that the average size of a FLOOD discovery message is about 1 Kbyte in both scenarios. It follows that CKDP messages tend to be much smaller than FLOOD discovery messages.

From these considerations, it turns out that although the CKDP protocol causes additional messages, the resulting overall additional traffic may be negligible with respect to the FLOOD discovery traffic. In details, FLOOD sends 144,580 bits in the dense scenario and 88,856 bits in the sparse one, while CKDP sends 9,792 bits in the dense scenario and 4,032 bits in the sparse one. Therefore, *NoB* is ≈ 6% in the dense scenario and ≈ 4% in the sparse one.

It turns out that while the additional round of messages required by CKDP causes the discovery phase in SeFLOOD to get longer than FLOOD, the additional SeFLOOD traffic results instead practically negligible with respect to the overall FLOOD traffic. Such a negligible traffic also makes the additional SeFLOOD energy overhead practically negligible with respect to the overall FLOOD energy overhead.

As to reconfiguration phase, SeFLOOD does not introduce any additional message with respect to FLOOD. SeFLOOD introduces some overhead in terms of *NoB* because they convey authenticators for integrity and proof of origin. Such an overhead is at most 1% (see Figure 14).

However, it is worthwhile to notice that CKDP assumes a random deployment according to which a node cannot know its neighbourhood until deployment is completed. However, and underwater acoustic network is often employed in mission-oriented systems such as the surveillance system considered in the UAN project. In such systems, nodes are initially located in predefined locations. Therefore, a node knows in advance its *potential* neighbours. Of course, depending on the sea conditions, a potential neighbour becomes an *actual* one if it can actually communicate with the node. If a node knows its *potential* neighbourhood in advance, then the first phase of CKDP, namely the discovery phase, can be completely avoided. Then, during the key distribution phase, the node is able to recognize its *effective* neighbors. This allows SeFLOOD to reduce the number of sent discovery messages and bits and makes the protocol faster.

### 7.3. On Packet Loss

Underwater acoustic networks are prone to packet loss. If we consider the packet loss (PL) as the ratio between the average number of lost packets and the number of sent packets, then PL = 1 − ADR (Section 7.1). It follows that in our experiments we experienced a packet loss that ranges from 32.8% to 61.5% (see Figure 10).

As it turns out, the problem of reliable communication is certainly important in UASNs but it is largely orthogonal to the problem of secure communication and thus we do not address it here. Interested readers can refer to the literature [1,38]. Notwithstanding, the security suite has been designed in such a way that it does not negatively interfere with reliable communication. In order to achieve this goal we have adopted three main solutions. First of all, we have limited the ciphertext expansion phenomenon by properly designing the cryptographic primitives (Section 5).

Second, we have used the User Datagram Protocol (UDP) in key management protocols of the security suite, namely, the Group Key Management Protocol (Section 5.3) and the Cluster Key Distribution Protocol (Section 6.3). In an underwater acoustic network, UDP presents several advantages with respect to connection-oriented protocols such as TCP. Mainly, UDP does not require an end-to-end connection, and therefore entirely avoids all the connection management issues that are typical of connection-oriented protocols and that cause them to perform poorly in a networking environment that is characterized by long delays and large losses. Of course, the main drawback of UDP is that it does not provide the same level of reliability in message delivery. Therefore, services such as packet re-transmission have to be moved to the upper layers. Here, it comes the third solution we have adopted.

As to the Group Key Management Protocol, if any node misses any of the rekeying messages then the node fails to receive the new group key *g*^+^. However, upon the next group communication, the node will be able to detect the missing event because it is not able to decrypt the received messages. The node can thus recover the key directly from the gateway GW.

Similarly, in the Cluster Key Distribution Protocol, if any node misses any key distribution message, the node fails to receive a cluster key *c*. However, upon the next cluster communication, the node will be able to detect the missing event because it is not able to decrypt the received messages. The node can thus recover the cluster key directly from the cluster head.

## 8. Conclusions

In this paper, we have presented a suite for secure underwater acoustic communication. The security suite comprises a routing protocol and a set of cryptographic primitives (cipher, digest, and re-keying), and has been designed to protect the confidentiality and the integrity while taking into account the unique characteristic and constraints of the underwater networking environment. Experiments and simulations have shown that the security suite introduces limited and sometimes negligible overhead, and thus it is perfectly adequate for the underwater acoustic communication. In particular, the suite achieves the following results.

- The cryptographic suite provides efficiency by limiting the effect of ciphertext expansion. The resulting overhead is indistinguishable from the performance fluctuations due to the changing sea conditions.
- The Discovery Phase of the secure routing protocol introduces an additional round, and thus a consequent delay, with respect to the insecure protocol. However it causes a negligible additional communication overhead (smaller than 6%). The overhead of the Discovery Phase is paid at system bootstrap and it is amortized over the whole system lifetime.
- The Reconfiguration Phase of the routing protocol does not introduce any additional overhead with respect to the insecure routing protocol. This is a very important feature because network reconfiguration due to node mobility, joining and leaving the application is a normal operational mode of the system.
- The design of the secure routing protocol meets the well-known Lampson's recommendations for computer systems design [39], while reconfiguration must be very efficient, it is sufficient that discovery is able to make progress provided it remains practically sustainable.

To the best of our knowledge, this is the first work that provides a complete, practical, and efficient solution to integrity and confidentiality for UASNs. In this respect, we hope that our work will have a significant impact in this field.

## Figures and Tables

**Table 1.** Configuration parameters for Scenario L.

**Node**	**Distance from GW (m)**	**Depth (m)**

GW	-	90.3
FN1	163.5	96
FN2	860	39
FLG1	677.8	20
FLG2	687.1	15

**Table 2.** Simulation parameters.

**Parameter**	**Value**	

Duration	5,000 s.	
Number of runs	15	
Average transmission range	2 km	
Maximum transmission power	189 dB re *μ*Pa	
Depth	200 m	
Number of nodes	9	
DIFS (silent period before transmission is considered)	0.25 s	
SIFS (Wait time between DATA and ACK)	0.01 s	
Max number of retransmissions	7	

	**Sparse Scenario**	**Dense Scenario**

Average number of Neighbors	2	5
